# Effect of inter-cycle interval on oocyte production in humans in the presence of the weak androgen DHEA and follicle stimulating hormone: a case-control study

**DOI:** 10.1186/1477-7827-12-68

**Published:** 2014-07-21

**Authors:** David H Barad, Vitaly A Kushnir, Ho-Joon Lee, Emanuela Lazzaroni, Norbert Gleicher

**Affiliations:** 1 The Center for Human Reproduction, 21 East 69th St, New York, New York, USA; 2 Foundation for Reproductive Medicine, New York, New York, USA

**Keywords:** Dehydroepiandrosterone (DHEA), In vitro fertilization (IVF), Diminished ovarian reserve (DOR), Oocyte yield, Follicle stimulating hormone (FSH), Gonadotropin s, Anti-Müllerian hormone (AMH)

## Abstract

**Background:**

In various animal models androgens have been demonstrated to enhance follicle stimulating hormone (FSH) activity on granulosa cells during small growing follicle stages. To assess whether similar synergism may also exist in humans we investigated women on androgen (dehydroepiandrosterone, DHEA) supplementation with varying concomitant FSH exposure.

**Methods:**

In a case controlled cohort study we determine if time interval between IVF cycles of IVF treatment with FSH had an effect on ovarian response to ovulation induction in women supplemented with DHEA. Among 85 women with known low functional ovarian reserve (LFOR), supplemented with DHEA, and undergoing at least 3 consecutive IVF cycles, 68 demonstrated short (<120 days) intervals between repeated cycles (Group 1) and were, therefore, considered to have consistent FSH exposure. In contrast 17 women (Group 2) demonstrated long (> = 120 days) intervals between repeated cycles and, therefore, were considered to demonstrate inconsistent FSH exposure. Trends in oocyte yields were compared between these groups, utilizing mixed model repeated measures ANOVA, adjusted for initial age and FSH dose.

**Results:**

Only women in Group I demonstrated a linear increase in oocyte yields across their three cycles of treatments (F = 7.92; df 1, 68.6; p = 0.017). Moreover, the analysis revealed a significant interaction between the two patient groups and cycle number for retrieved oocytes (F = 6.32, df = 2, 85.9, p = 0.003).

**Conclusions:**

This study offers preliminary confirmatory evidence that repeated short interval exposure to androgens in combination with FSH improves human FOR. A higher level of evidence will require prospectively randomized studies.

## Background

Androgens are increasingly recognized as potentially beneficial for follicle maturation [1]. Increasing androgen receptors appear on granulosa cells of human follicles from transitional to primary and secondary follicle stages [2]. Granulosa cell specific androgen receptor knockout (ARKO) mice have provided evidence of reduced follicle progression and increased follicle atresia compared to wild type mice at these early stages of follicle maturation [3]. The androgen receptor at those early stages of follicle development, thus, appears to play a critical role in normal mammalian follicle development.

It has also been reported that in rodents at least one function of androgens at these early follicle stages is increasing the sensitivity of granulosa cells to follicle stimulating hormone (FSH) [4,5]. It thus appears that androgens and FSH at these early follicle stages act synergistically. Whether this also applies to the human experience is, however, unknown.

Investigators have recently started to integrate androgen supplementation into fertility therapy. Lisi et al., for example, used LH priming in attempts to increase androgen production during folliculogenesis, reporting improved embryo grades and implantation rates [6]. The currently most widely clinically utilized androgen is dehydroepiandrosterone (DHEA), which has been associated with an improvement of a variety of outcomes, especially in women with low functional ovarian reserve (LFOR) [7].

The small growing follicle pool represents the individual’s functional ovarian reserve (FOR). Potential effects of androgen/FSH supplementation are on the growing follicle pool and, as a result, need to be administered for at least six weeks [1], though benefits of DHEA have been observed to be cumulative up to approximately four to five months [8]. Best pregnancy results are obtained if DHEA supplementation is combined with in vitro fertilization (IVF) [7,8], and results of DHEA supplementation directly correlate with how well DHEA converts to testosterone [9].

Whether the above described androgen/FSH synergism can further augment beneficial effects on small growing follicles of androgens alone, is, however, unknown. Based on anecdotal patient experiences, we hypothesized that such androgen/FSH synergism can be assumed more likely if repeated ovulation induction cycles in close proximity were to produce more oocytes than repeated cycles with more intercycle distance.

The objective of this study was, therefore, to determine whether time intervals between IVF cycles in women on DHEA supplementation, indeed, demonstrated such an effect.

## Methods

We identified 140 women from the center’s electronic research data bank who were supplemented with DHEA, and underwent at least three consecutive IVF cycles. To assess potential effect of repeated FSH exposure while using DHEA, we subdivided these patients into those with repeated IVF cycles within 120 days of each other (Group 1, n = 68) and those with cycles that were spaced at greater than 120-day intervals (Group 2, n = 72). A 120-day interval was chosen as cut off because this is approximately the time for a small growing follicle to move from primary to antral stage follicle [10]. Small growing follicle stages are the stage in which FSH/androgen synergism in animal models was demonstrated [3].

Women who failed three consecutive IVF cycles in close proximity usually demonstrated severely diminished ovarian reserve. Women with long inter-cycle intervals, however, were more mildly affected since some, indeed, had long intervals between IVF cycles because of an intervening pregnancies. Women selected for Group 2, therefore, were further matched for age and level of ovarian reserve, based on FSH and anti-Müllerian hormone (AMH) levels, and women with an intervening pregnancy between IVF cycles were excluded from analysis. In all, 55 women were, therefore, excluded because of pregnancy or failure to meet matching criteria for age and ovarian reserve. This left for final analysis a comparison of 68 patients in Group 1 and only 17 patients in Group 2.

Once diagnosed with LFOR, patients at our center receive a uniform supplementation protocol with 25 mg of pharmaceutical grade, micronized DHEA, TID, uninterrupted until pregnancy (second, normally rising human chorionic gonadotropin level) or termination of fertility treatment attempts with autologous oocytes [7].

After at least six weeks of DHEA supplementation, a first IVF cycle is initiated with the patient’s first menses. All LFOR patients receive identical ovarian stimulation in a microdose agonist protocol (leuprolide acetate, Lupron™, Abbot Pharmaceuticals, North Chicago, IL, USA), as previously reported [7]. Ovarian stimulation always involves a preponderance of 300 to 450 IU of FSH (products of various manufacturers, depending on patient preference and insurance circumstances) and 150 IU of a human menopausal gonadotropin (hMG) product (mostly, Repronex™, Ferring Pharmaceuticals, Parsippany-Troy Hills, NJ, USA).

IVF cycle outcomes were assessed in every patient’s first, second and third cycle, based on oocyte yields, cancellation rates and number of embryos transferred.

Statistical analysis was performed using SPSS version 21.0 (IBM SPSS, Chicago IL).

Chi-square tests were used to compare proportions. Continuous variables were presented as means ± standard deviations (SD), and tested either by Student’s t-test and/or analysis of variance. Changes in oocyte production across cycles were evaluated using a general linear model for repeated measures. Linear Mixed models with repeated measures were used to test the effects of age and total gonadotropin dosage as covariates, with a factor identifying sets of cycles, based on </≥120 day intervals between cycles. The Linear Mixed Model provides an F statistic that allows us to make an inference about the observed effect. Degrees of freedom (df) were calculated using Satterthwaite approximation. All tests were two-tailed, and a P-value of P < 0.05 was considered statistically significant.

This study involved only analyses of data derived from our center’s anonymized research database. All of the center’s patients sign at initial presentation an informed consent, which allows utilization of medical records for research purposes, as long as confidentiality of medical records and anonymity of the patient is maintained. Both conditions were met for this study. Under these conditions the study qualified for expedited approval from the center’s Institutional Review Board study approval # ER010813-01 Jan 13, 2013.

Pregnancy rates, quite obviously, could not be compared between the two groups since patient selection criteria in this study only allowed for pregnancies to occur in 3rd cycles.

## Results

Patient characteristics and primary infertility diagnoses are listed in Table 1. The primary infertility diagnosis of all women was LFOR. As the table demonstrates, mean age, body mass index (BMI), FSH, AMH and primary infertility diagnoses of both groups were similar. Mean oocyte yields across all cycles were also similar between groups, 2.87 ± 3.03 for the group 1 and 3.49 ± 3.23 for the group 2.

**Table 1:** Patient characteristics

**Cycle interval**	**< 120 days**	**≥ 120 days**
N	68	17
Age (years)	40.50 ± 4.45	41.59 ± 3.16
BMI (kg/m2)	23.73 ± 3.9	23.00 ± 4.07
FSH (mIU/mL)	19.6 ± 17.9	16.7 ± 21.4
AMH (ng/mL)	0.42 ± 0.41	0.54 ± 0.40
Gonadotropin dosage/IVF cycle	6720 ± 2403	6209 ± 2646
Oocyte yield/IVF cycle	2.87 ± 3.03	3.49 ± 3.23
**Race (n/%)**		
Caucasian	49 (72%)	10 (65%)
African	10 (15%)	3 (12%)
Asian	9 (13%)	4 (24%)
**Diagnosis (n/%)**		
Male factor	13 (18.1%)	4 (23.5%)
Endometriosis	4 (5.6%)	1 (5.9%)
PCO	0 (0.0%)	0 (0.0%)
DOR	55 (76.4%)	11 (64.7%)
Tubal	12 (16.7%)	2 (11.8%)
Uterine	5 (6.9%)	1 (5.9%)

The two patient groups did not differ significantly in any listed characteristics.

Repeated measures ANOVA confirmed that in Group 1 age (p < 0.001), FSH dose (p = 0.05), oocytes retrieved (p = 0.001) and embryos transferred (p = 0.004) significantly increased across the three cycles, while percentages of cycles with no oocytes retrieved (p = 0.024) and canceled cycles (p = 0.006) decreased across the three cycles of treatment for Group 1. The number of canceled cycles increased for Group 2 (p = 0.028) (Table 2). Although FSH dose increased across the three cycles, in Group 1, there was no significant interaction between FSH dosage and cycles of treatment with respect to number of oocytes retrieved. This observation suggests that the observed cycle to cycle trend for oocytes retrieved was not related to the small difference in FSH dosage observed between the two groups.

**Table 2:** Oocyte yields in 1st - 3rd cycles among patients who completed three cycles of treatment with < or ≥120 days interval between cycles

	**N**	**Cycles of treatment**			**P (linear trend)**
		**1st cycle**	**2nd cycle**	**3rd cycle**	
**Group 1 - Time < 120 days**					
*Age (years)*	68	40.2 ± 4.4	40.4 ± 4.3	40.5 ± 4.3	0.001
*FSH Dose (units)*	68	6519 ± 2202	6486 ± 2344	7381 ± 2355	0.05
*No Oocytes*	68	23 (33.8)^a^	17 (25.0)	11 (16.2)^a^	0.024
*Oocyte (total)*	68	2.81 (2.02 - 3.60)^b,c^	3.84 (2.88 - 4.80)^b^	4.50 (3.48 - 5.53)^c^	0.001
*Embryos transferred*	68	1.10 (0.78-1.42)^b^	1.37 (0.99-1.74)	1.59 (1.2-1.95)^b^	0.004
*Cancelled Cycles*	68	32.4% (23–42)^d^	18% (8–27)	13% (4–23)^d^	0.006
**Group 2 - Time ≥ 120 days**					
*Age (years)*	17	41.6 ± 3.2	42.2 ± 3.0	42.5 ± 3.2	0.001
*FSH Dose (units)*	17	6965 ± 2712	6520 ± 2288	5070 ± 2714	0.08
*No Oocytes*	17	2 (11.8)	4 (23.5)	5 (29.4)	0.22
*Oocyte (total)*	17	4.35 (2.78 – 5.92)	3.35 (1.78 - 4.93)	2.77 (1.19 – 4.33)	0.185
*Embryos transferred*	17	1.24 (0.62-1.85)	1.06 (0.44-1.67)	0.82 (0.21-1.44)	0.60
*Cancelled Cycles*	17	0	0	18% (7–29)	0.028

Values are presented as means (95% confidence intervals) or ± standard deviation.

^a^p = 0.03; ^b^p = 0.003; ^c^p = 0.001; ^d^p = 0.018.

A linear mixed model with repeated measures, adjusted for the effects of age and FSH dosage administered during ovulation induction, was used to test for an interaction of the effect of short vs. long cycle intervals across three sequential cycles on trend in oocyte production. In this model a highly significant interaction was seen (F = 6.32, df = 2, 85.9, p = 0.003) between repeated cycles of treatment and short vs. long cycle interval, indicating a positive increase in oocyte numbers from cycle to cycle in Group 1 but no significant increase in Group 2.

Because of this observed interaction the change in retrieved oocytes across the three cycles was in subsequent analyses evaluated separately for short and long interval treatment groups.

Repeated measures ANOVA, adjusted for age and FSH dose, revealed a linear increase in oocyte yields for women with short inter-cycle intervals < 120 days (Group 1) across the three cycles of treatments (F = 7.92; df 1, 68.6; p = 0.017), while women with cycle interval of ≥ 120 days (Group 2) did not demonstrate such an effect.

**Figure 1:**
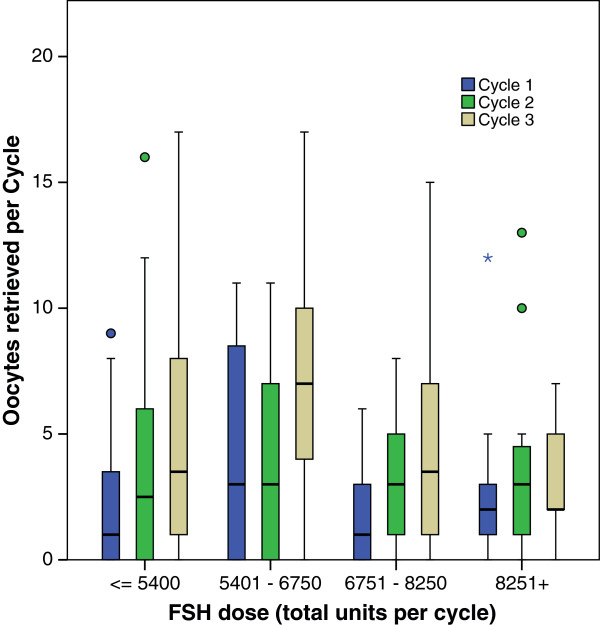
**Total gonadotropin -dosages used in 3 cycles in Group 1.** The figure demonstrates increases in oocyte yields from 1st to 3rd IVF cycle in Group 1 patients independent of gonadotropin dosage administered.

**Figure 2:**
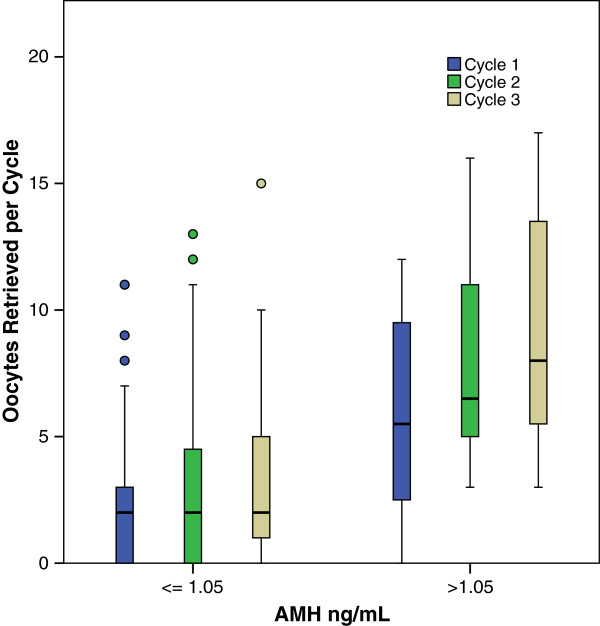
**Oocyte numbers retrieved in Group 1.** Patients in 3 cycles depending on AMH level cut off 1.05 ng/mL. This figure demonstrates increases in oocyte yields between 1st and 3rd cycle independent of the woman’s AMH levels

To better understand the possible effects of gonadotropin dosage and of levels of ovarian reserve, we performed further analyses of Group 1: Oocyte yields in Group 1 increased from cycle to cycle independently of gonadotropin dosage administered for ovulation induction (Figure 1). Subdividing Group 1 patients further into poor and good prognosis patients, based on an AMH cut off of 1.05 ng/mL, representing, independent of age, the cut-off AMH value that discriminates between better and poorer IVF pregnancy chances [11], the increase in oocyte yields was similar in both sub-groups (Figure 2).

The definition of both study groups did not allow for assessment of pregnancies in 1st and 2nd IVF cycles since, by patient selection criteria, they had to be zero. There were 4 clinical pregnancies in Group 1 patients (5.9%) in their third treatment cycle. In contrast, Group 2 patients experienced no pregnancies in third cycles (n.s.).

## Discussion

While there has been an ongoing concern that repeated cycles of ovulation induction might deplete ovarian reserve [12-14], existing evidence suggests that in most cases ovarian yields in IVF are constant across repeated cycles [15]. This study offers evidence that among women with severe LFOR, using androgen supplementation with DHEA, oocyte yields increase across repeated cycles when the interval between cycles is 120 days or less (Table 2). This suggests a potential functional synergism between androgens and FSH/gonadotropin supplementation in women with LFOR.

Clinically, the observed increased oocyte yield suggests that patients with LFOR who fail to achieve pregnancy in one cycle may benefit from closely scheduled consecutive IVF cycles, taking advantage of a possible DHEA/FSH synergism. Such an approach is contradictory to current practice patterns, which favor breaks between cycles reserve [12-14].

Animal data strongly suggest synergistic effects of androgens and FSH at small growing follicle stages [3]. Whether such effects are, however, also present in humans is unknown. We, therefore, looked for a model that would allow us to make inferences about androgen/FSH interaction at early stages of follicle maturation. We hypothesized that consecutive IVF cycles, if close enough together, should offer relatively steady FSH exposure. This idea arose from observations of our center’s DHEA index patient, who had undergone nine consecutive IVF cycles, month after month, demonstrating evidence of steadily improving FOR, despite significantly advancing age during cycle progression [16].

Previous studies found an association of numbers of small growing follicles (i.e., FOR) with IVF outcomes [17-19]. As Table 2 demonstrates, closely spaced ovulation induction cycles with repeated FSH exposure in parallel to DHEA supplementation significantly increased oocyte yields from cycle to cycle, while absence of steady FSH exposure results in no significant change in oocyte numbers. Moreover, the positive effects observed in Group 1 were similar at all severity levels of LFOR, whether defined by gonadotropin stimulation dosage (Figure 1) or AMH levels (Figure 2), supporting the idea that this increase represents synergism between androgens and gonadotropin s.

A diagnosis of LFOR was in this study reached based on age-specific ovarian reserve (OR) criteri [20,21]. Highly abnormal FSH levels of 19.6 ± 17.9 and 16.7 ± 21.4 mIU/mL and AMH levels of 0.42 ± 41 ng/mL and 0.54 ± 0.40 (Table 1), respectively, clearly indicate how adversely patients in this population were affected.

The observed decline in oocyte number in Group 2 was only nominal. It, nevertheless, was surprising since we expected to find no change in oocyte numbers in this group over three consecutive cycles. The data in this group, however, demonstrate an increase in cycle cancellations in third cycles. Since canceled cycles statistically were treated as “0” oocytes, this observation may partially explain the nominal decline. Furthermore, longer cycle intervals, of course, also lead to proportionally greater reproductive aging, although each of these analyses was also adjusted for age.

We previously reported that an AMH value of 1.05 ng/mL discriminates at all ages between better and poorer IVF pregnancy chances in women with LFOR [11]. While there remains some debate among experts concerning the best AMH cut-off for prediction of pregnancy [11,22-25], patients with higher AMH will respond more effectively to ovulation induction than those with lower AMH levels [22,26-28]. Therefore, we further subdivided our already initially adversely selected patients into women with more severe DOR (AMH ≤ 1.05 ng/mL) and those with milder DOR and better IVF prognosis (AMH >1.05 ng/mL). As Figure 2 demonstrates, both categories of AMH performed quite similarly across the three cycles of treatment among Group 1 patients, suggesting that androgen/FSH synergism is effective at all severity levels of LFOR.

### Strengths and limitations

Strength of this analysis is that the population studied is comprised of an unusually large number of women with LFOR, undergoing repeated cycles of IVF. Furthermore, study participants in both groups were very carefully matched.

As noted in Table 2 we did observe a nominal increase in gonadotropin dosage across the three cycles of treatment in Group 1, and a nominal decrease in gonadotropin dosage in Group 2. We adjusted for cycle gonadotropin dosage in all of our models. It, however, is possible that such adjustments may not fully correct for an effect of differences in gonadotropin dosage. Though quite unlikely, we, therefore, cannot completely rule out that observed differences in ovarian response, at least in part, could be due to these differences in gonadotropin doses.

Epsteiner et al. [29] reported increased oocyte yields across repeated cycles among women with normal ovarian reserve, however the cycle interval in that study was allowed to vary up to one year. That study found that increase in gonadotropin dosage was not associated with an increase in oocyte yield among women with diminished ovarian reserve.

While it is also possible that the observed change in oocyte yield over the course of three observations represents regression to the mean, the clear difference in trend of oocytes retrieved across cycles was observed using a repeated measures analysis that examined the change in oocyte production across cycles of treatment. This statistical analysis was not dependent on absolute difference in oocytes retrieved per cycle between each group. Furthermore, for Group 1 an increase in oocytes from cycle to cycle was seen at all levels of gonadotropin use, and among both, severe and less severe cases of LFOR. In contrast, there was no evidence of increased oocyte production among patients in Group 2.

The principal weakness of this analysis is that it is based on retrospective data. To obtain a higher level of evidence for synergism between androgens and gonadotropin s in a clinical setting would require a prospective randomized controlled trial with DHEA/placebo, fixed gonadotropin dosages and fixed long and short inter-cycle intervals.

Given the retrospective design and small numbers of patients it is not possible to draw conclusion about the effect of treatments on pregnancy chances. It, nevertheless is remarkable that in Group 1, even in 3rd cycles, pregnancies were still established, once more confirming that with appropriate treatment, pregnancies can still be established in women frequently believed by many to be beyond help with utilization of autologous oocytes.

## Conclusions

While other authors have reported no change in oocyte yields in consecutive IVF cycles, except for decreases attributed to advancing female age [30-32], this study demonstrates that oocyte yields under androgen/FSH supplementation continue to increase through three closely-spaced consecutive IVF cycles. This observation suggests that women with severe LFOR, who despite limited pregnancy chances, wish to pursue IVF with autologous oocytes, should, at least, consider three consecutive cycles. Whether here observed rise in oocyte yield continues with additional time of supplementation exposure to androgen/FSH is not known.

This study further suggests the possibility of new ovarian stimulation protocols specifically directed at early stages of follicle maturation. Such a protocol could use low dose gonadotropin preparations along DHEA supplementation for two to three months to increase testosterone levels, advancing small follicle cohorts into gonadotropin -sensitive stages of follicle maturation, at which point a routine stimulation could be initiated.

## Abbreviations

(AMH): Anti-Müllerian hormone; (ARKO): Androgen receptor knockout; (BMI): Body mass index; (CHR): Center for Human Reproduction; (DHEA): Dehydroepiandrosterone; (FOR): Functional ovarian reserve; (FSH): Follicle stimulating hormone; (HMG): Human menopausal gonadotropin; (IVF): In vitro fertilization; (LFOR): Low functional ovarian reserve; (ns): Not significant, ovarian.

## Competing interests

DHB has received research and grant support, travel funds, and speaker honoraria from various pharmaceutical and medical device companies, not related to topics presented in this article; is listed as an inventor on four already-awarded and other still-pending U.S. patents claiming beneficial effects in diminished ovarian reserve (DOR) and embryo ploidy from dehydroepiandrosterone (DHEA) or from other androgen supplementations and has received patent royalties from this company; is listed as co-inventor on a number of pending U.S. patents claiming diagnostic relevance for the assessment of triple CGG repeats on the FMR1 gene in determining risk toward DOR and related issues. VAK has nothing to disclose. H-JsL has nothing to disclose. EL has nothing to disclose. N.G. is owner of CHR, where this research was conducted and owns shares in Fertility Nutraceuticals, LLC, a producer of fertility supplements, and has received patent royalties from this company; has received research and grant support, travel funds, and speaker honoraria from various pharmaceutical and medical device companies, not related to topics presented in this article; is listed as a inventor on four already-awarded and other still-pending U.S. patents claiming beneficial effects in diminished ovarian reserve (DOR) and embryo ploidy from dehydroepiandrosterone (DHEA) or from other androgen supplementations; is listed as co-inventor on a number of pending U.S. patents claiming diagnostic relevance for the assessment of triple CGG repeats on the FMR1 gene in determining risk toward DOR and related issues. A full listing of all patent information can be provided on request.

## Authors’ contributions

DHB conceived, designed and performed analysis and interpretation of the data, drafted, revised and approved the final manuscript. VAK participated in acquisition and interpretation of data, assisted in revision of the manuscript and gave final approval. H-J.L, EL each assisted with acquisition of data, assisted in critical revision of the manuscript and gave final approval. NG participated in design of the study, data acquisition and interpretation of data, assisted in critical revision of the manuscript and gave final approval. All authors read and approved the final manuscript.
